# Exploring the Association Between Sleep Patterns, Pubertal Health, and Phthalate Exposure—Preliminary Results from Slovakia

**DOI:** 10.3390/toxics13040286

**Published:** 2025-04-08

**Authors:** Martina Jahnátková, Henrieta Hlisníková, Ida Petrovičová, Branislav Kolena

**Affiliations:** 1 Department of Zoology and Anthropology, Constantine the Philosopher University in Nitra, 949 74 Nitra, Slovakia; 2 Institute of Environmental Medicine, Karolinska Institutet, Nobels Väg 13, 171 77 Stockholm, Sweden

**Keywords:** phthalates, sleep quality, puberty, endocrine disruptors, biomonitoring

## Abstract

Background: This study aims to explore the association between sleep patterns in children and their exposure to phthalates to assess potential health implications. Methods: Participants (n = 60) completed the Pittsburgh Sleep Quality Index (PSQI) questionnaire. Consumer behavior scores (CBS) were calculated. The Tanner scale was used to monitor the stages of puberty. First-morning urine samples were analyzed by high-performance liquid chromatography–tandem mass spectrometry. Results: The average sleep duration was 8 h and 44 min, with boys sleeping significantly longer (*p* = 0.01). Notably, 51.7% of participants reported sleeping less than 9 h. The nonlinear effects of phthalate metabolite in association with PSQI were observed for hydroxy-mono-isononyl phthalate (OH-MiNP, *p* = 0.003) and MnOP (*p* < 0.001), indicating that the relationship does not follow a simple linear pattern. Simple linear regression revealed a significant positive association between the Mono-n-octyl phthalate (MnOP) and PSQI scores (*p* = 0.016). After adjustment for place of residence, BMI, CBS, sex, and age, the significance of associations between phthalate metabolites and sleep quality diminished, necessitating cautious interpretation. No statistically significant associations between pubertal changes and the value of PSQI as well as phthalates were observed. Conclusion: Our results provide preliminary evidence of potential nonlinear associations that require validation in a larger cohort. The findings highlight the importance of monitoring phthalate exposure in children, as it may influence sleep patterns and overall health.

## 1. Introduction

The relationship between sleep patterns, exposure to environmental factors, and children’s health is complex and multifaceted.

Sleep duration during sensitive periods of child development is crucial for emotional, behavioral, and cognitive outcomes [1,2]. A study found that children with shorter nighttime sleep had higher BMI z-scores, indicating a link between sleep and adiposity [3].

Melatonin, cortisol, and insulin are pivotal in regulating human sleep patterns. Melatonin is crucial for synchronizing the circadian rhythm, primarily produced in the pineal gland, and its levels rise in the evening to promote sleep onset [4,5]. It enhances sleep quality and regulates various physiological processes, including immune function and body temperature [5,6]. Cortisol exhibits a diurnal rhythm, peaking in the morning and declining throughout the day. Elevated evening cortisol levels can disrupt sleep onset and quality [7,8]. Studies indicate that poor sleep correlates with increased cortisol levels, suggesting a bidirectional relationship between sleep and stress responses [8]. Insulin’s role in sleep is less direct but significant; it influences glucose metabolism and can affect sleep quality [6,8]. Insulin regulates blood glucose levels that are essential for normal brain function during sleep; disruptions in insulin activity can lead to hypoglycemia or hyperglycemia, causing sleep disturbances such as awakening, restless sleep, or frequent urination, thereby negatively impacting sleep quality.

Sleep is crucial for the secretion of growth hormones (GHs), vital for children’s growth and metabolism. Interestingly, acute sleep disruptions do not appear to diminish GH secretion, indicating some resilience in the endocrine response despite sleep fragmentation [9]. The importance of sleep quality is shown by several studies focusing on sleep disruption in hospitalized probands. These studies show that hospitalized children experienced significant sleep disruption due to environmental factors such as noise and light, which can hinder recovery and overall health [8,10].

Research indicates that some of the environmental factors, such as endocrine-disrupting chemicals (EDCs), directly and significantly impact endocrine function [11,12]. Since sleep is, among others, others closely linked to changes in hormone concentrations, it is appropriate to investigate the effects of substances with endocrine-disrupting properties. A study found that exposure to EDCs like phthalates and bisphenol A correlates with reduced sleep duration, particularly in individuals with vitamin D deficiency, suggesting a compounded negative effect on sleep health [13].

Phthalates, commonly found in various consumer products, have been linked to sleep disturbances. Research indicates that exposure to phthalates in sleeping environments, particularly in university dormitories, is prevalent [14]. Furthermore, studies have shown that higher phthalate exposure correlates with reduced sleep efficiency, increased sleep latency among pregnant women [15], as well as heightened sleep problems in younger adult females [16]. Adolescents also face risks, with certain phthalate metabolites associated with shorter sleep duration [17]. In midlife women, phthalate exposure has been linked to increased sleep disturbances, suggesting a complex interaction between environmental toxins and sleep quality [18].

While the evidence points to a negative impact of phthalates on sleep, it is essential to consider that individual susceptibility and lifestyle factors may also play significant roles in these associations. The objective of this study is to monitor sleep patterns using the standardized PSQI questionnaire (Pittsburgh Sleep Quality Index), in association with phthalate biomonitoring from first-morning urine samples and an assessment of the consumer behavior of children aged 10–15 years.

## 2. Materials and Methods

The research phase of this study was conducted between October 2022 and December 2022. The target group consisted of socially and ethnically homogeneous primary school students from Cabaj-Čápor (Nitra District, Slovakia; n = 60; 100% response rate), with an age range of 10 to 15 years. The research was carried out under the project “Biomonitoring of Endocrine Disruptors and Selected Parameters in the Slovak Population in the Framework of Research in the Physiological-Analytical Laboratory,” reviewed and approved by the Ethics Committee of Constantine the Philosopher University in Nitra on 25 October 2022 (UKF-2022/1191-2:191013). Participation in this study, and consent for sample collection, analysis, and processing were provided by the child’s legal guardian through written informed consent. The exclusion criteria for study participation comprised a positive response to questions monitoring diagnosed medical conditions, failure to provide a first-morning urine specimen, or incomplete responses to any questionnaire items. These stringent criteria were implemented to maintain data integrity and minimize confounding variables throughout the analytical process.

A trained professional completed a standardized questionnaire at the participants’ homes in the presence of the parent and child, monitoring sleep quality and quantity (PSQI), health status, and consumer behavior (Supplementary Materials Supplement 1). In the study, three 2 mL urine samples were collected from each participant on a single day, recognizing that while a multi-day collection would have provided a more comprehensive representation of long-term exposure trends, logistical constraints and the non-compensated nature of participation made a one-day collection the most viable approach. Urine samples were stored in a cooling box before being transferred to storage and later analyzed at the Physiological Analytical Laboratory. All samples and data were processed blindly.

### 2.1. Anthropometric Methods

Body height (cm) and weight (kg) were measured using standard anthropometric methods. Based on the obtained absolute values, relative values were calculated using the body mass index (BMI, kg/m^2^) online calculator.

### 2.2. Consumer Behavior Score

Based on participants’ responses pertaining to consumer behavior monitoring, which are included in Supplement 1 (comprising food consumption patterns, cosmetics usage, and consumer behavior practices), we conducted the consumer behavior score (CBS) calculation. This comprehensive assessment incorporated all reported consumption patterns to derive a quantitative metric of exposure-relevant behaviors. The responses were converted into point values using a Likert scale, and the score was obtained by summing these values and dividing them by the number of monitored items.

### 2.3. Assessment of Physical Changes Associated with Puberty Onset

The monitoring of physical changes associated with puberty onset (in the context of the thelarche, pubarche, menarche, and genital development) was performed using the standardized Tanner scale [19].

### 2.4. Sleep Monitoring

The participants were given the standardized Pittsburgh Sleep Quality Index questionnaire, which assessed their usual sleep habits over the past 30 days [20].

### 2.5. Urinary Phthalate Analyses

First-morning urine samples (2 mL per sample) were collected in phthalate-free Eppendorf microtubes, transported in a cooling box, and stored in a deep freezer at −74 °C until analysis. The details of the qualitative and quantitative analysis of phthalate metabolites have been described in a previous study [21]. The urinary concentrations of phthalate metabolites were measured using high-performance liquid chromatography (HPLC) coupled with tandem mass spectrometry (MS/MS) (Infinity 1260 and 6410 triple quad, Agilent, Santa Clara, CA, USA), following published HPLC-MS/MS online methods [21,22]. The analyses were conducted in the Physiological Analytical Laboratory, which participated in the HBM4EU QA/QC program. Z-scores for these compounds ranged from −1.1 to 0.7, within the acceptable range of ≤|2| as required for successful interlaboratory testing [23]. Internal quality control was conducted by analyzing two control materials (a mixture of urine samples) with known low and high concentrations. The limits of quantification (LOQ) were between 1 and 2.5 ng/mL.

### 2.6. Statistics

Descriptive statistics, including means, medians, standard deviations (SDs), and percentiles, were calculated for all continuous variables. The normality of the data was assessed using the Shapiro–Wilk test. To compare continuous variables between boys and girls, independent samples *t*-tests were utilized for normally distributed data (age, height, and consumer behavior score [CBS]), while the Mann–Whitney U test was employed for non-normally distributed variables (weight, BMI, PSQI scores, and phthalate metabolites). Correlations between phthalate metabolites and PSQI scores were examined using Pearson or Spearman correlation coefficients, depending on the data distribution. To evaluate the impact of phthalate metabolite concentrations on sleep quality, linear regression analyses were conducted with log-transformed phthalate metabolites as predictor variables, PSQI scores as the dependent variable, and BMI, residence, age, and CBS as confounders, identified from our knowledge and prior experience with the data. Model fit was assessed using the coefficient of determination (R^2^). Measured phthalate metabolite concentrations were categorized into tertiles to assess potential nonlinear associations with sleep quality parameters. The study population was divided into three equal groups (tertiles A, B, and C) based on the distribution of each phthalate metabolite concentration. Tertile A represented the lowest exposure group (0–33rd percentile), tertile B comprised the medium exposure group (34th–66th percentile), and tertile C included the highest exposure group (67th–100th percentile). This approach allowed for the examination of concentration-dependent relationships while minimizing the impact of extreme values. Additionally, the Kruskal–Wallis test was applied to compare the PSQI scores across terciles of phthalate metabolite concentrations, followed by pairwise comparisons to identify significant differences between specific terciles. A significance level of *p* < 0.05 was set for determining statistical significance. Analyses were performed using the statistical software Jamovi (version 2.2) [24].

## 3. Results

The average age reached 12.67 ± 1.32 years with no significant sex differences. Most participants (91.67%) come from rural areas, and 91.67% report exposure to passive smoking. The baseline characteristics in Table 1 show various factors in a cohort of 60 participants (51.67% boys; 47.33% girls). The results suggest some minor sex-dependent differences. Girls (49.74 ± 11.70 kg) tend to be heavier than boys (45.72 ± 13.35 kg), though this difference is on the border of statistical significance (*p* = 0.07). Consumer behavior scale (CBS) levels are notably higher in girls than boys (0.42 ± 0.1 vs. 0.31 ± 0.1; *p* ≤ 0.001).

**Figure 1:**
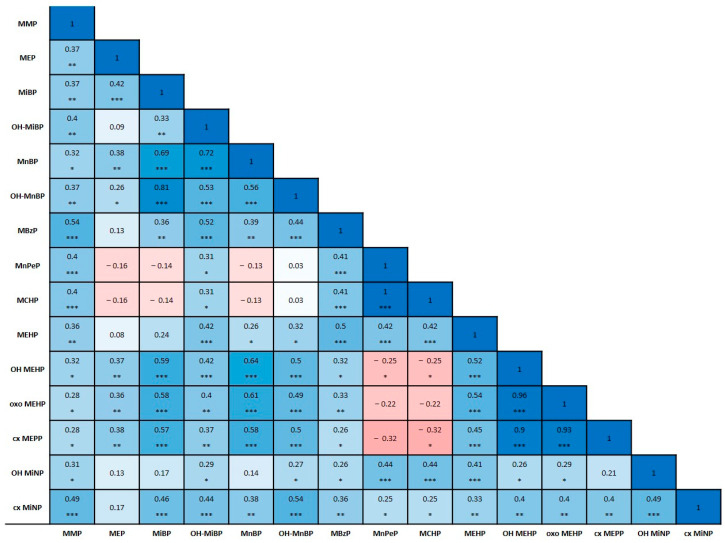
Correlation between phthalate metabolites in the cohort (n = 60). Notes: MMP—Mono-methyl phthalate; MEP—Mono-ethyl phthalate; MiBP—Mono-isobutyl phthalate; OH-MiBP—Mono(hydroxy-iso-butyl) phthalate; MnBP—Mono-n-butyl phthalate; OH-MBP—Mono(hydroxy-n-butyl) phthalate; MBzP—Mono-benzyl phthalate; MnPeP—Mono-n-pentyl phthalate; MCHP—Mono-cyclohexyl phthalate; MEHP—Mono-2-ethylhexyl phthalate; OH-MEHP—Mono(2-ethyl-5-hydroxyhexyl) phthalate; oxo-MEHP—Mono(2-ethyl-5-oxohexyl) phthalate; cx-MEPP—Mono(2-ethyl-5-carboxypentyl) phthalate; OH-MiNP—Hydroxy-mono-isononyl phthalate; cx-MiNP—Carboxy-mono-isononyl phthalate; * *p* < 0.05, ** *p* < 0.01, *** *p* < 0.001; red color indicates a negative correlation, blue color indicates a positive correlation; color intensity reflects the strength of the correlation.

The correlation between phthalate metabolites in the cohort is illustrated in Figure 1. The correlation matrix highlights strong positive and negative relationships between certain metabolites, which suggest interconnected metabolic pathways and potential co-exposure patterns.

**Figure 2:**
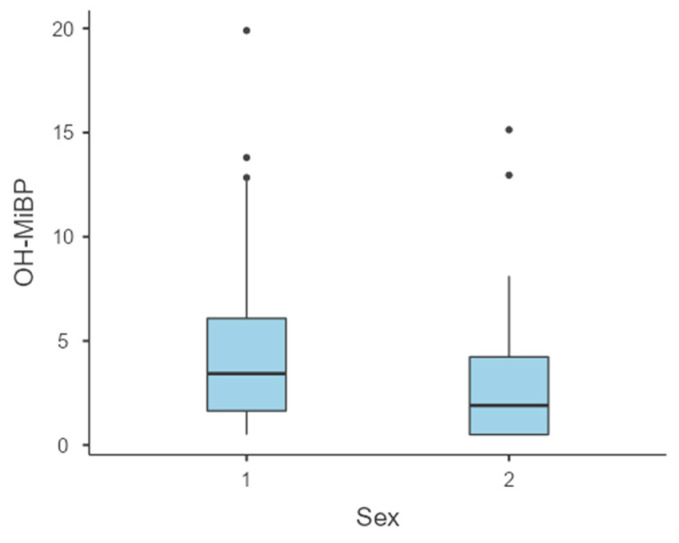
Sex-dependent differences in concentration of OH-MiBP metabolites (ng/mL). Notes: x-axis: sex (1—boys, 2—girls); y-axis: concentration of hydroxy-mono-isobutyl phthalate (OH-MiBP); dots—Outliers; Horizontal line in box—Median (50th percentile); Whiskers—Data range (excluding outliers); Box—Interquartile range.

Findings underscore the importance of considering the cumulative exposure to multiple phthalates, as their interactions may amplify their biological effects. Although higher concentrations of phthalate metabolites were observed in boys in addition to MEP and cx-MiNP, a statistically significant difference in concentration was recorded only for the metabolite OH-MiBP (Table 1, Figure 2).

The average time of falling asleep (hh:mm:ss) was 21:29:42; girls went to bed later than boys, although this difference was not statistically significant (21:33:07 ± 00:53:08 vs. 21:24:29; *p* = 0.279). The average time of waking up was 06:39:30; boys woke up later, but the difference was not statistically significant (06:43:12 ± 00:33:59 vs. 06:36:00 ± 00:31:24; *p*= 0.24. The average length in minutes required to fall asleep (mm:ss) was 24:08—although the sex-related difference was huge, we did not observe a statistically significant difference between the sexes (boys 17:58 ± 03:23 vs. girls 31:03 ± 00:27, *p* = 0.085).

The average sleep duration for the entire cohort was 8:44 ± 0.97 h. Boys had a significantly longer sleep duration (average = 8.74 ± 0.95 h; median = 9 h) than girls (average = 8.13 ± 0.98 h; median = 8 h, *p* = 0.01). Furthermore, 51.7% of participants reported sleeping less than the recommended 9 h, while 23.33% reported sleeping less than 8 h daily.

The overall sleep quality, as measured by the PSQI, is consistent across sexes. The average score is 1.65 for boys and 1.62 for girls, with minimal variation in the interquartile range. The range of scores spans from 1.00 to 8.00 for both, showing some variability in individual sleep experiences, though this variation is relatively small. The difference between boys and girls is not statistically significant (*p* = 0.618), suggesting no meaningful sex-based disparity in sleep quality within this cohort. We observed a negative correlation between MnOP and PSQI (r = 0.39, *p* = 0.002).

In the linear regression analysis (Table 2), the model fit measures indicate a moderate association between log-transformed phthalate metabolites and the outcome of the PSQI score (R = 0.431), with 18.6% of variance in the PSQI score (R^2^ = 0.186). Among the phthalate metabolites analyzed, only MnOP showed a statistically significant association with Pittsburgh Sleep Quality Index (PSQI) scores. While the crude analysis showed a significant relationship (β = 10.1557, *p* = 0.016), this association did not persist after controlling for potential confounders, including place of residence, BMI, CBS, sex, and age. All other metabolites indicate that they do not appear to meaningfully influence sleep quality based on this analysis.

**Figure 3:**
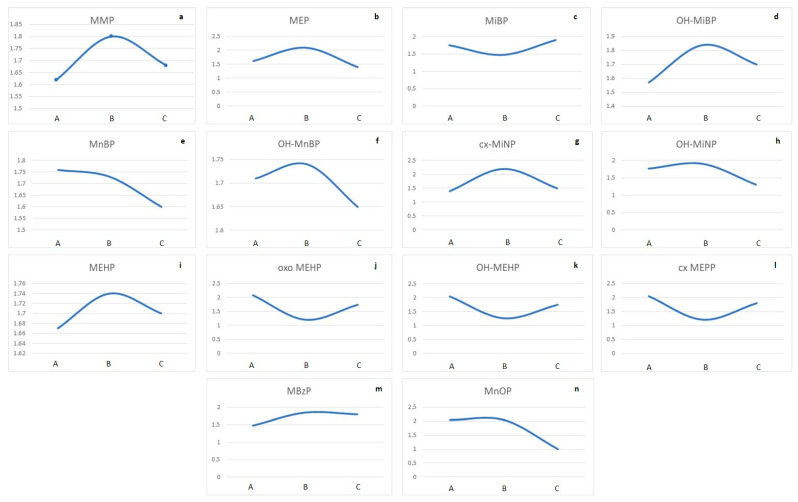
Trend of phthalate metabolites distribution (ng/mL) and potential effect on PSQI score (Notes: x-axis: A—first tercile of phthalate metabolite concentration (ng/mL); B—second tercile of phthalate metabolite concentration (ng/mL); C—third tercile of phthalate metabolite concentration (ng/mL); (**a**). MMP—Mono-methyl phthalate; (**b**). MEP—Mono-ethyl phthalate; (**c**). MiBP—Mono-isobutyl phthalate; (**d**). OH-MiBP—Mono(hydroxy-iso-butyl) phthalate; (**e**). MnBP—Mono-n-butyl phthalate; (**f**). OH-MnBP—Mono(hydroxy-n-butyl) phthalate; (**g**). cx-MiNP—Carboxy-mono-isononyl phthalate; (**h**). OH-MiNP—hydroxy-mono-isononyl phthalate; (**i**). MEHP—Mono-2-ethylhexyl phthalate; (**j**). oxo-MEHP—Mono(2-ethyl-5-oxohexyl) phthalate; (**k**). OH-MEHP—Mono(2-ethyl-5-hydroxyhexyl) phthalate; (**l**). cx-MEPP—Mono(2-ethyl-5-carboxypentyl) phthalate; (**m**). MBzP—Mono-benzyl phthalate; (**n**). MnOP—Mono-n-octyl phthalate; y-axis: PSQI score.

The nonlinear effects of phthalate metabolites (ng/mL) concerning the PSQI score were observed (Figure 3a–n).

To examine how varying levels of exposure to phthalate metabolites may relate to sleep quality as measured by the PSQI and illuminate potential associations with sleep disturbances, highlighting the need for further investigation into the health implications of these environmental chemicals, we employed the Kruskal–Wallis test (Table 3).

The results suggest that there are significant differences in the concentrations of OH-MiNP and MnOP among the groups in this study. Based on an inter-tercile distribution comparison of phthalate metabolites and the PSQI score, we observed a statistically higher concentration of the metabolite OH MiNP in the second tercile (B) compared to the first tercile (A; *p* = 0.003), and a statistically significantly higher concentration of MnOP in the second tercile (B) compared to the first tercile (A), and comparing the first tercile (A) with the third tercile of MnOP (C; *p* <  0.001). The effect sizes for the metabolites vary, with MnOP showing a strong effect (ε^2^ = 0.595), followed by OH-MiNP (ε^2^ = 0.199), indicating that these metabolites have a substantial impact concerning the PSQI. All pairwise comparisons for non-significant associations can be found in the Supplementary Materials Supplement 2.

**Figure 4:**
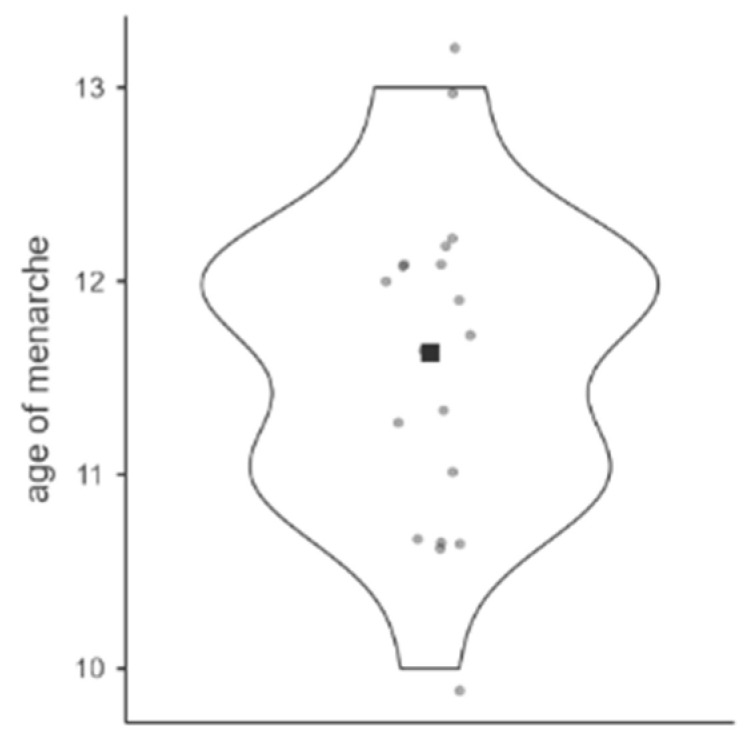
Menstrual onset age distribution (years). Notes: Violin plot showing the distribution of age at menarche. Dots represent individual observations, while the width of the “violin” indicates the frequency of occurrence at a given age.

Pubertal changes were evaluated in boys and girls using Tanner staging. The data indicate that most participants, both girls and boys, are in the early stages of puberty, with only a small proportion advancing to later stages. Menarche had occurred in 67.9% of female participants; most girls reported the onset of menarche at age 12 (47.37%, Figure 4), and the average age of menarche reached 11.63 ± 0.73 years.

For the thelarche, the majority are in the early stages, with 31% in stage 2 and 31% in stage 3. A smaller percentage, 27.6%, are in stage 4, and only 6.9% have reached stage 5, indicating that most girls are in the earlier phases of breast development. For the pubarche in girls, 34.5% are in stage 2 and 31% in stage 3, with 20.7% still in stage 1. Only 6.9% of girls have reached stages 4 and 5, showing that most are in the initial stages of pubic hair development.

The pubarche and genital development of boys show that 39.3% are in stage 2, and 32.1% remain in stage 1. A smaller group, 17.9%, is in stage 3, while only a few boys have reached the more advanced stages, with 7.1% in stage 4 and 3.6% in stage 5.

In female participants, we observed associations approaching statistical significance between thelarche and the concentrations of the metabolites oxo-MEHP and cx-MEPP (*p* = 0.074) and OH-MiNP (*p* = 0.072). A borderline significant association was identified between OH-MiNP metabolite levels and genital development in male participants (*p* = 0.055). In all the aforementioned cases, no significant associations were found between phthalate levels in pairwise comparisons or during analyses of other pubertal changes. In the cohort, based on the results of the Kruskal–Wallis test, we did not observe any associations between pubertal changes (in the context of thelarche, pubarche/and genital development, and menarche) and the value of the PSQI (Supplementary Materials Supplement 3). We did not observe a correlation between the age of the first menarche and the PSQI score (r = −0.11; *p* = 0.642).

## 4. Discussion

Sleep duration is defined as the period during which sleep occurs [25], and the quality of sleep can be influenced by a variety of behavioral, psychological, and pathophysiological factors [26], as well as environmental factors, among others, by endocrine disruptors. Inappropriate sleep patterns are associated with negative health outcomes. Recent research has highlighted the critical importance of sleep for mental health, revealing that the risk of developing depression doubles in sleep-deprived individuals [27]. Adolescents’ sleep patterns differ internationally and among various sociodemographic groups. On school days, the average sleep duration spans from 7 h and 47 min to 9 h and 7 min, while on non-school days, it ranges from 9 h and 31 min to 10 h and 22 min. The percentage of adolescents meeting sleep recommendations ranges from 32% to 86% on school days and from 79% to 92% on non-school days [28]. In our cohort, 51.7% of participants reported sleeping less than the recommended 9 h daily. A 2011 systematic review of 41 studies found later bedtimes in adolescents from Europe than from North America (22:46 vs. 22:06) and longer sleep on school days (8:26 vs. 7:28 h) [29]. In our study, we observed a discrepancy with this finding, as our respondents go to bed earlier and sleep longer. Our results do not match the results from some studies, which found that boys sleep less and go to bed later than girls [30,31,32]. On the other hand, our results comply with other studies, which found that boys sleep more in comparison to girls [33,34]. After comparing the average sleep time with data from countries monitored by Gariepy et al. [28], our measured value (8 h 44 min) was closest to the data recorded in Canada (8 h 41 min) and furthest from the data from Poland (7 h 47 min). Compared to the values from the monitoring period of 2013–2018 from Slovakia in this study, our established average sleep time was 37 min higher. Our results are consistent with the study mentioned above, in which girls woke up earlier than boys on school days (girls, 06:36 vs. boys, 06:43).

In the context of associations between sleep patterns, pubertal health, and phthalate exposure observed in our study, primarily, we must emphasize that the limited sample size restricted our ability to conduct complex adjusted regression models. In contrast, the variability between unadjusted and adjusted results underscores the significance of covariates when evaluating the relationships between phthalate metabolites and sleep quality. The adjusted linear regression results indicate that the initially identified associations may align with a null effect, highlighting the necessity for larger cohort studies. Consequently, the findings presented herein should be interpreted within the context of the aforementioned methodological constraints, and extrapolation beyond this study population should be approached with appropriate caution regarding external validity.

A study conducted on Mexican teenagers found that higher levels of exposure to endocrine-disrupting chemicals are linked to both later and longer sleep durations, with different EDCs contributing to these effects [35]. Specifically, the study identified that various phthalates—MBzP, oxo-MEHP, OH-MEHP, and MEP—significantly influenced the impact of combined EDC exposures on sleep patterns. In our study, we identified in the unadjusted model a positive significant association between MnOP and the PSQI (*p* = 0.016); however, it became non-significant after adjusting for a place of residence, BMI, CBS, sex, and age. This association implies that higher MnOP concentrations might be linked to better sleep quality outcomes, while other metabolites do not show a meaningful impact on sleep quality.

Furthermore, we observed the nonlinear effects of phthalate metabolites concerning PSQI scores, suggesting that the association between exposure and outcome is not monotonic, with adverse effects present at both low and high exposure levels. This phenomenon has been documented in other health-related outcomes in animal and human toxicological studies globally, not only concerning phthalates [36,37] but also other endocrine-disrupting chemicals [38,39]. In this context, results indicated significant differences in OH-MiNP and MnOP concentrations among study groups. These findings underline the necessity for further research into the health implications of environmental chemicals on sleep quality, in which a bigger sample size will allow a more exact examination of the cumulative effect of phthalates and other chemical substances, with an emphasis on their often-conflicting effects in the context of sleep patterns.

These findings partially align with previous research that demonstrated a correlation between elevated levels of certain phthalates and modified sleep patterns in adolescents [17]. Additionally, animal studies have shown that prenatal exposure to other EDCs, such as bisphenol A, negatively affects brain volume in children and young rats, as observed through magnetic resonance imaging (MRI) [40]. Further research indicates that EDCs can disrupt the suprachiasmatic nucleus (SCN), leading to disturbances in circadian rhythms in animals. This disruption involves both endocrine hormones and the genes regulating circadian rhythms [41]. In a study from 2024, exposure to MEP and MBzP has been shown to exacerbate the negative effects of vitamin D deficiency, particularly in reducing sleep duration [13]. Exposure to phthalates, chemicals commonly found in plastics, personal care products, and household items, has been linked to sleep problems in children. Recent studies suggest that higher levels of phthalate metabolites in the body are associated with increased sleep disturbances, including difficulty falling asleep and decreased sleep duration. Phthalates may disrupt endocrine function, potentially altering melatonin production and affecting circadian rhythms [41,42]. In our study, a borderline significant association (*p* = 0.0554) was found between the levels of the OH-MiNP metabolite and genital development in boys. Although this association does not reach conventional significance, it merits attention within the context of ongoing investigations into the effects of environmental endocrine disruptors, such as phthalates, on pubertal development [43]. These findings underscore the importance of minimizing phthalate exposure to promote better sleep health in the pediatric population.

Puberty represents a critical physical and hormonal change period, marked by progressive development observable through Tanner staging in both boys and girls. Our results suggest that the majority of participants are going through the early stages of puberty, with a notable predominance of the girls experiencing menarche, with the reported average age of onset at 11.63 years. This timing aligns with the existing literature, which typically mentions a range of onset for menarche between ages 10 and 15, noting that environmental and genetic factors can influence this timing [44,45]. It corresponds well with data on thelarche and pubarche, revealing that many are still in the initial phases of breast and pubic hair development. The distribution of 31% of participants in thelarche stages 1 and 2 indicates that these physical changes are beginning relatively early, consistent with recent studies illustrating that many girls are experiencing these developments at younger ages than previous generations [46]. The proportions observed in this cohort emphasize the importance of continued research into the factors contributing to variations in the timing and progression of puberty.

Similarly, in boys, the distribution of pubarche and the genital development stages suggests a predominance at earlier developmental stages, with 37.9% categorized in stage 1. This observation is indicative of the overarching trends identified in male pubertal development, reflecting a progressive maturation that may be modulated by a multitude of biological and environmental factors [47,48].

Studies have suggested that endocrine-disrupting xenobiotics can interfere with hormonal pathways, potentially impacting the timing, progression, or discrepancies of puberty [48,49]. However, this current evaluation did not find significant associations between phthalate levels and other aspects of pubertal changes, indicating a need for further research to elucidate these complex relationships.

A relatively small sample size (n = 60) could limit the statistical power and generalizability of the findings. The participants came from a socially and ethnically homogeneous group, which may not be representative of broader populations. The cross-sectional design of this study prevents establishing causal relationships between phthalate exposure and sleep patterns. This study relied on self-reported sleep data monitored by standardized PSQI questionnaires, which could introduce recall bias. Additionally, the analysis of phthalate exposure was based on single time point urine samples, which may not accurately reflect long-term exposure patterns given that phthalate metabolites have short half-lives. This study also did not control for all potential confounding factors influencing sleep patterns, such as screen time, or other environmental exposures.

This study’s novelty lies in its focus on a vulnerable developmental period, offering new insights into participants’ challenges. It also provides an analysis of non-monotonic effects, which adds significant value to our understanding of how these challenges may affect sleep patterns.

## 5. Conclusions

This study offers preliminary insights into potential associations between phthalate metabolites and sleep quality, although model adjustments indicate results that may align with the absence of an association. Consistent with prior studies, we found that phthalate exposures can potentially alter sleep patterns, including sleep duration and increased sleep disturbances. These findings emphasize the need for minimizing phthalate exposure to promote better sleep outcomes and overall well-being. Despite methodological limitations concerning sample size, our findings could lay the groundwork for further research involving larger cohorts and more robust analytical methods. Future studies with enhanced statistical power should validate the nonlinear relationships identified in this work through adjusted models to confirm or refute the actual existence of associations. Conclusions should be interpreted with consideration of the cross-sectional nature and methodological limitations that hinder generalization to broader populations.

## Figures and Tables

**Table 1:** Baseline characteristics of the cohort.

	Sex	n	%	Mean	Median	SD	Minimum	Maximum	25th Percentile	75th Percentile	*p*
Age (years)	all	60	100	12.67	12.00	1.32	10.00	15.00	11.00	13.00	0.190
	boys	31	51.67	12.00	12.00	1.18	10.00	15.0	11.00	13.00	
	girls	29	48.33	12.83	13.00	1.43	10.00	15.00	11.00	13.00	
Residence	rural	55	91.67								
	urban	5	8.33								
Passive smoking	yes	55	91.67								
	no	5	8.33								
Weight (kg)	all			47.66	44.00	12.63	29.00	87.00	39.50	53.10	**0.070**
	boys			45.72	42.00	13.35	29.00	87.00	39.00	45.50	
	girls			49.74	51.00	11.70	32.00	83.00	42.00	56.00	
Height (cm)	all			156.29	157.50	10.43	130.00	185.00	151.00	162.00	0.228
	boys			154.71	153.00	11.06	135.00	185.00	149.00	160.00	
	girls			157.98	160.00	9.62	130.00	175.50	154.00	163.00	
BMI (kg/m^2^)	all			19.35	18.19	4.05	13.850	33.98	16.87	21.19	0.387
	boys			18.83	18.08	3.73	14.609	33.98	16.84	19.95	
	girls			19.91	18.51	4.36	13.85	32.42	17.26	22.86	
CBS	all			0.36	0.33	0.12	0.17	0.70	0.27	0.43	**≤0.001**
	boys			0.31	0.30	0.10	0.17	0.60	0.23	0.33	
	girls			0.42	0.37	0.11	0.27	0.70	0.33	0.50	
PSQI Score TOTAL	all			1.00	1.00	1.65	1.00	8.00	1.00	1.00	0.618
	boys			1.65	1.00	1.76	1.00	8.00	1.00	1.50	
	girls			1.62	1.00	1.55	1.00	8.00	1.00	1.00	
MMP (ng/mL)	all			79.88	7.46	526.75	1.77	4224.400	3.24	14.16	0.953
	boys			9.26	7.75	7.84	1.77	32.64	3.82	13.61	
	girls			164.98	7.36	781.36	1.77	4224.400	3.16	20.77	
MEP (ng/mL)	all			218.37	15.18	775.31	0.00	5464.44	6.46	46.59	0.998
	boys			144.75	18.56	377.28	3.71	1771.09	7.59	31.31	
	girls			319.56	14.84	1075.56	3.32	5464.44	6.47	73.42	
MiBP (ng/mL)	all			43.03	26.76	57.56	0.00	343.44	14.99	45.55	0.436
	boys			41.81	30.72	33.45	8.10	137.04	16.17	50.84	
	girls			48.70	22.70	76.89	2.53	343.44	16.27	38.94	
OH-MiBP (ng/mL)	all			4.16	2.54	4.84	0.50	21.67	1.05	4.77	**0.053**
	boys			4.72	3.43	4.65	0.50	19.90	1.64	6.08	
	girls			3.15	1.90	3.68	0.50	15.13	0.50	4.23	
MnBP (ng/mL)	all			51.20	35.07	47.34	0.00	201.76	21.12	63.16	0.121
	boys			61.46	40.80	51.03	11.14	201.76	27.81	81.13	
	girls			45.44	32.09	41.84	5.17	191.26	20.40	62.08	
OH-MnBP (ng/mL)	all			25.04	9.87	77.10	0.71	609.10	4.79	17.15	0.263
	boys			19.40	11.70	24.08	2.03	123.14	6.97	18.42	
	girls			34.36	8.18	112.02	0.71	609.10	4.83	13.63	
MBzP (ng/mL)	all			2.45	1.23	5.18	0.71	31.67	0.71	2.47	0.229
	boys			1.10	1.46	1.47	0.71	7.37	1.01	2.50	
	girls			2.37	1.16	4.22	0.71	20.45	0.71	1.62	
MnPeP (ng/mL)	all			3.05	1.25	14.26	1.25	100.00	1.25	1.25	LOQ
	boys			1.00	1.25	0.00	1.25	1.25	1.25	1.25	
	girls			1.00	1.25	0.00	1.25	1.25	1.25	1.25	
MCHP (ng/mL)	all			2.22	0.50	14.39	0.50	100.00	0.50	0.50	LOQ
	boys			0.50	0.50	0.00	0.50	0.50	0.50	0.50	
	girls			0.50	0.50	0.00	0.50	0.50	0.50	0.50	
MEHP (ng/mL)	all			4.16	3.42	2.02	1.41	9.51	2.77	5.31	0.427
	boys			4.80	3.44	2.15	2.00	9.51	2.95	5.70	
	girls			4.03	3.56	1.85	1.83	8.76	2.56	4.81	
OH MEHP (ng/mL)	all			11.43	9.65	9.11	0.00	46.64	5.74	15.99	0.111
	boys			14.50	12.75	10.64	1.72	46.64	6.22	19.59	
	girls			10.00	8.76	6.08	1.26	25.86	5.96	14.35	
oxo MEHP (ng/mL)	all			9.18	8.18	6.36	0.00	34.52	5.06	11.58	0.117
	boys			10.71	10.26	6.60	2.14	34.52	5.66	14.29	
	girls			8.85	7.84	5.61	1.65	29.28	5.14	9.65	
cx MEPP (ng/mL)	all			17.85	14.11	13.04	0.00	70.91	10.37	23.85	0.089
	boys			21.08	18.37	13.19	5.30	70.91	11.57	27.84	
	girls			16.22	13.27	11.96	2.93	60.59	9.31	19.31	
OH MiNP (ng/mL)	all			4.18	0.75	12.28	0.75	76.70	0.75	2.01	0.562
	boys			4.05	0.75	13.55	0.75	76.70	0.75	2.03	
	girls			1.85	0.75	2.29	0.75	12.82	0.75	1.73	
cx MiNP (ng/mL)	all			15.74	5.85	57.30	0.71	457.65	1.55	13.30	0.197
	boys			23.70	5.75	81.07	0.71	457.65	2.09	15.35	
	girls			8.26	5.95	14.25	0.71	77.29	0.71	8.66	
MnOP (ng/mL)	all			2.95	0.00	14.43	0.00	100.00	0.00	0.75	0.474
	boys			0.22	0.00	0.35	0.00	0.75	0.00	0.75	
	girls			0.29	0.00	0.37	0.00	0.75	0.00	0.75	

Notes: Except age, height and CBS (tested by *t*-test), all data were tested employed the Mann–Whitney test; BMI—body mass index in kg/m^2^; CBS—consumer behavior score; PSQI Score—score of Pittsburgh Sleep Quality Index; MMP—Mono-methyl phthalate; MEP—Mono-ethyl phthalate; MiBP—Mono-isobutyl phthalate; OH-MiBP—Mono(hydroxy-iso- butyl) phthalate; MnBP—Mono-n-butyl phthalate; OH-MnBP—Mono(hydroxy-n-butyl) phthalate; MBzP—Mono-benzyl phthalate; MnPeP—Mono-n-pentyl phthalate; MCHP—Mono-cyclohexyl phthalate; MEHP—Mono-2-ethylhexyl phthalate; OH-MEHP—Mono(2-ethyl-5-hydroxyhexyl) phthalate; oxo-MEHP—Mono(2-ethyl-5-oxohexyl) phthalate; cx-MEPP—Mono(2-ethyl-5-carboxypentyl) phthalate; OH-MiNP—hydroxy-mono-isononyl phthalate; cx-MiNP—Carboxy-mono-isononyl phthalate; Bold formatting indicates statistical significance.

**Table 2:** Results of linear regression log-transformed phthalate metabolites and PSQI score.

			95% CI				
Predictor	β	SE	Lower	Upper	*t*	*p*	−β
MMP log	−0.395	0.493	−1.388	0.598	−0.8012	0.427	−0.13997
MEP log	0.2623	0.383	−0.51	1.035	0.6841	0.497	0.11893
MiBP log	0.82	2.278	−3.767	5.407	0.36	0.72	0.19988
OH-MiBP log	0.6898	1.476	−2.284	3.663	0.4672	0.643	0.19401
MnBP log	0.3191	2.342	−4.398	5.036	0.1363	0.892	0.06637
OH-MnBP log	−0.3733	1.615	−3.626	2.879	−0.2312	0.818	−0.1074
MBzP log	0.0783	0.816	−1.565	1.722	0.0959	0.924	0.0161
MEHP log	0.5654	1.638	NaN	NaN	0.3452	0.732	0.0658
OH MEHP log	1.7087	2.286	NaN	NaN	0.7475	0.459	0.3466
oxo MEHP log	−3.8417	4.024	−2.734	3.865	−0.9548	0.345	−0.63197
cx MEPP log	0.0519	2.901	−2.896	6.313	0.0179	0.986	0.00876
OH MiNP log	−0.3687	0.845	−11.945	4.262	−0.4364	0.665	−0.08334
cx MiNP log	−0.0893	0.656	−5.792	5.895	−0.1361	0.892	−0.03206
MnOP log	10.1557	4.041	−2.071	1.333	2.5131	**0.016**	0.3656

Note: The estimate represents the magnitude and direction of the relationship between the predictor variable and the dependent variable; SE (standard error); the *t*-value—dividing the estimate by its standard error; MMP—Mono-methyl phthalate; MEP—Mono-ethyl phthalate; MiBP—Mono-isobutyl phthalate; OH-MiBP—mono(hydroxy-iso-butyl) phthalate; MnBP—Mono-n-butyl phthalate; OH-MnBP—Mono(hydroxy-n-butyl) phthalate; MBzP—Mono-benzyl phthalate; MEHP—Mono-2-ethylhexyl phthalate; OH-MEHP—Mono(2-ethyl-5-hydroxyhexyl) phthalate; oxo-MEHP—Mono(2-ethyl-5-oxohexyl) phthalate; cx-MEPP—Mono(2-ethyl-5-carboxypentyl) phthalate; OH-MiNP—hydroxy-mono-isononyl phthalate; cx-MiNP—Carboxy-mono-isononyl phthalate; Bold formatting indicates statistical significance.

**Table 3:** Kruskal–Wallis comparison of the inter-tercile distribution of phthalate metabolites (ng/mL) to the PSQI score.

	χ^2^	*p*	ε^2^
MMP	0.307	0.858	0.00521
MEP	0.427	0.808	0.00724
MiBP	0.401	0.818	0.0068
OH-MiBP	0.182	0.913	0.00309
MnBP	1.04	0.595	0.0176
OH-MnBP	0.174	0.917	0.00295
MBzP	0.185	0.912	0.00314
MEHP	0.27	0.874	0.00458
OH-MEHP	0.18	0.527	0.0.0217
oxo MEHP	3.05	0.217	0.0518
cx MEPP	1.38	0.501	0.0234
OH-MiNP	11.7	**0.003**	0.199
	A-B	**0.004**	
	A-C	0.125	
	B-C	0.193	
cx MiNP	4.3	0.116	0.0729
MnOP	35.1	**<0.001**	0.595
	A-B	**<0.001**	
	A-C	**<0.001**	
	B-C	NaN	

Notes: A—first tercile of phthalate metabolite concentration (ng/mL); B—second tercile of phthalate metabolite concentration (ng/mL); C—third tercile of phthalate metabolite concentration (ng/mL); MMP—Mono-methyl phthalate; MEP—Mono-ethyl phthalate; MiBP—Mono-isobutyl phthalate; OH-MiBP—Mono(hydroxy-iso-butyl) phthalate; MnBP—Mono-n-butyl phthalate; OH-MnBP—Mono(hydroxy-n-butyl) phthalate; cx-MiNP—Carboxy-mono-isononyl phthalate; OH-MiNP—hydroxy-mono-isononyl phthalate; MEHP—Mono-2-ethylhexyl phthalate; oxo-MEHP—Mono(2-ethyl-5-oxohexyl) phthalate; OH-MEHP—Mono(2-ethyl-5-hydroxyhexyl) phthalate; cx-MEPP—Mono(2-ethyl-5-carboxypentyl) phthalate; MBzP—Mono-benzyl phthalate; MnOP—Mono-n-octyl phthalate; Bold formatting indicates statistical significance.

## Data Availability

The data presented in this study are available on request from the corresponding author.

## Supplementary Materials

The following supporting information can be downloaded at: https://www.mdpi.com/article/10.3390/toxics13040286/s1, Supplement 1: Questionnaire: Exploring the Link Between Sleep Patterns, Pubertal Health, and Phthalate Exposure in Slovak Youth; Supplement 2: Kruskal-Wallis comparison of the inter-tercile distribution of phthalate metabolites (ng/mL) to PSQI score; Supplement 3: Kruskal-Wallis comparison of the pubertal changes (in the stage 1–5, scaled by Tenner) to PSQI score.

## Abbreviations

The following abbreviations are used in this manuscript:

PSQI	Pittsburgh Sleep Quality Index
CBS	Consumer behavior score
HPLC	High-performance liquid chromatography
MS/MS	Tandem mass spectrometry
BMI	Body mass index
MnOP	Mono-n-octyl phthalate
OH-MiNP	Hydroxy-mono-isononyl phthalate
GH	Growth hormone
EDCs	Endocrine-disrupting chemicals
UKF	Constantine the Philosopher University in Nitra
HBM4EU QA/QC	European Human Biomonitoring Initiative—Quality Assurance/Quality Control
LOQ	Limit of quantification
SD	Standard deviation
p	Statistical significance
R	Person’s correlation coefficient
R^2^	Coefficient of determination
n	Frequency
MMP	Mono-methyl phthalate
MEP	Mono-ethyl phthalate
MiBP	Mono-isobutyl phthalate
OH-MiBP	Mono(hydroxy-iso-butyl) phthalate
MnBP	Mono-n-butyl phthalate
OH-MnBP	Mono(hydroxy-n-butyl) phthalate
MBzP	Mono-benzyl phthalate
MnPeP	Mono-n-pentyl phthalate
MCHP	Mono-cyclohexyl phthalate
MEHP	Mono-2-ethylhexyl phthalate
OH-MEHP	Mono(2-ethyl-5-hydroxyhexyl) phthalate
oxo-MEHP	Mono(2-ethyl-5-oxohexyl) phthalate
cx-MEPP	Mono(2-ethyl-5-carboxypentyl) phthalate
cx-MiNP	Carboxy-mono-isononyl phthalate
hh	Hours
mm	Minutes
ss	Seconds
β, −β	Regression coefficient
SE	Standard error
t	Value for hypothesis testing of β
log	Logarithm
ng	Nanogram
mL	Milliliter
χ^2^	Chi-squared test
ε^2^	Eta-squared
MRI	Magnetic resonance imaging
