# Supplementary material for: Exploring the Association Between Sleep Patterns, Pubertal Health, and Phthalate Exposure—Preliminary Results from Slovakia
Source: Toxics. 2025 Apr 8;13(4):286. doi: 10.3390/toxics13040286 (PMC12031374; doi:10.3390/toxics13040286)
Supplement: Supplementary file 1 [file toxics-13-00286-s001.zip › Supplement 1 Questionnaire and informed consent.pdf]

Exploring the Link Between Sleep Patterns, Pubertal Health, and Phthalate Exposure in Slovak Youth

Questionnaire and informed consent

Date of examination: **ID:**

The information contained in this questionnaire serves the needs of scientific research. The results are subject tofull anonymity. By submitting this questionnaire, I confirm that I agree to the processing of the information contained in the questionnaire below, as well as to the collection and processing of biological samples and anthropometric and sociometric data for the needs of scientific and statistical research.

....

Signature of the interviewer Signature of the proband/ child's legal guardian

---

1. **Sex: male** ☐ **female** ☐

2. **Weight:** .... **Height**: ....

3. **What is your ethnic/national origin:**

Slovak ☐ Hungarian ☐ Roma ☐ Czech ☐ other..................

4. **What diseases have you been diagnosed with**

thyroid disease	
diseases of the genital organs	
diseases of the hypothalamic-pituitary system	
other endocrine disease	
osteoporosis	
high blood pressure	
diabetes	
increased cholesterol	
cardiovascular problems	
respiratory problems	
neurobehavioral (ADHD)	
allergy (if yes, specify)	

If other, specify:

5. **Place of permanent residence:** urban ☐ rural ☐

6. **During the previous 24 hours, did you eat food heated in a plastic container or packaging?**
yes ☐ no ☐

7. **Did you drink a liquid from a plastic bottle or cup during the previous 24 hours?**
yes ☐ no ☐

8. **Indicate which of the following foods you have consumed in the last 48 hours:**

☐ margarine (Flora, Rama.......)
☐ sliced cheeses (slices in plastic wrap)
☐ salami (slices inplastic wrap)
☐ chilled, frozen meat products (in plastic packaging)
☐ baguettes, sandwiches, cakes (semi-finished products in plastic packaging)
☐ salads (Parisian, Italian...in plastic wrap)
☐ wafers, cookies, candies (in plastic wrap)
☐ chocolate spreads (nutella, nugget...)
☐ canned soups

9. **Indicate which of the following liquids you consumed in the last 48 hours:**

☐ water in plastic bottles (mineral, sweetened)
☐ milk, acidophilic drinks (in plastic bottles)
☐ hot drink (in a plastic bottle, or cup
☐ drinks in a can

10. **Did you have food you consumed within 48 hours stored in a plastic container (box)?**
yes ☐ no ☐

11. **What cosmetics have you used in the last 48 hours?**
- ☐ cream
- ☐ antiperspirant
- ☐ perfume
- ☐ nail polish
- ☐ body lotion, butter
- ☐ eye shadows, lipsticks, make-up
- ☐ shower gel
- ☐ hair shampoo
- ☐ only organic cosmetics
- ☐ antibacterial gel
- ☐ lip balm

12. **Have you used wet wipes in the last 24 hours?**
yes ☐ no ☐

**13. In what time interval before sleeping did you last use electronic devices?**	
**14. How much time did you spend using electronic devices before sleep?**	
**15. Is the room where you sleep exposed to artificial light sources during sleep?**	
**16. Do you have a darkened room when you sleep?**	
**17. In what time interval before sleep did you consume your last meal?**	
**18. Did you consume fatty and sugary foods before sleeping?**	
**19. Did you exercise in the afternoon?**	
**20. Did you engage in physical activity 3-4 hours before sleeping?**	
**21. Did you drink energy drinks 4-6 hours before sleeping?**	
**22. What is the usual temperature in your bedroom?**	
**23. Do you ventilate your bedroom before sleeping?**	
**24. Do you watch TV in the bedroom?**	
**25. Do you work on a computer in the bedroom?**	
**26. Do you read in the bedroom?**	
**27. Do you use the bedroom exclusively for sleeping?**	
**28. Do you go to bed and wake up at the same time?**	
**29. How much time do you spend on afternoon rest?**	

30. **In the image below, please circle the level of sexual characteristic development of your child.**

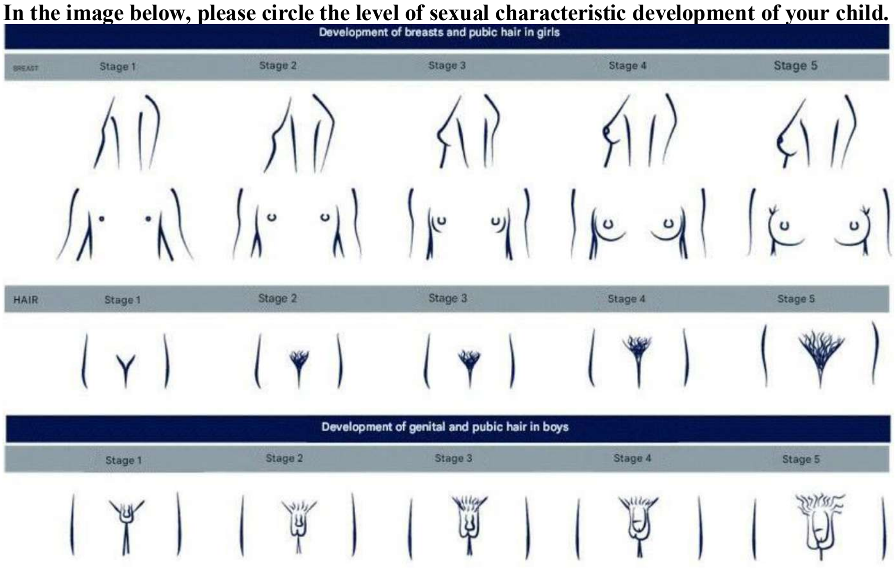

31. **Please provide the date of menarche:**
