# Supplementary material for: Exploring the Association Between Sleep Patterns, Pubertal Health, and Phthalate Exposure—Preliminary Results from Slovakia
Source: Toxics. 2025 Apr 8;13(4):286. doi: 10.3390/toxics13040286 (PMC12031374; doi:10.3390/toxics13040286)
Supplement: Supplementary file 1 [file toxics-13-00286-s001.zip › Supplement 2.pdf]

Kruskal-Wallis comparison of the inter-tercile distribution of phthalate metabolites (ng/ml) to PSQI score

	χ^2		df	p	ε^2
**MMP**	0.307		2	0.858	0.00521
		A-B		0.886	
		A-C		0.871	
		B-C		1	
**MEP**	0.427		2	0.808	0.00724
		A-B		0.804	
		A-C		0.955	
		B-C		0.915	
**MiBP**	0.401		2	0.818	0.0068
		A-B		0.856	
		A-C		0.999	
		B-C		0.854	
**OH-MiBP**	0.182		2	0.913	0.00309
		A-B		0.956	
		A-C		0.991	
		B-C		0.908	
**MnBP**	1.04		2	0.595	0.0176
		A-B		0.91	
		A-C		0.797	
		B-C		0.571	
**OH-MnBP**	0.174		2	0.917	0.00295
		A-B		0.986	
		A-C		0.966	
		B-C		0.908	
**MBzP**	0.185		2	0.912	0.00314
		A-B		0.951	
		A-C		0.986	
		B-C		0.916	
**MEHP**	0.27		2	0.874	0.00458
		A-B		0.926	
		A-C		0.879	
		B-C		0.997	
**OH-MEHP**	1.28		2	0.527	0.0217
		A-B		0.507	
		A-C		0.946	
		B-C		0.693	
**oxo MEHP**	3.05		2	0.217	0.0518
		A-B		0.183	
		A-C		0.837	
		B-C		0.455	
**cx MEPP**	1.38		2	0.501	0.0234
		A-B		0.495	
		A-C		0.951	
		B-C		0.643	
	11.7		2	**0.003**	0.199

**OH-MiNP**		A-B		**0.004**	
		A-C		0.125	
		B-C		0.193	
**cx MiNP**	4.3		2	0.116	0.0729
		A-B		0.21	
		A-C		1	
		B-C		0.21	
**MnOP**	35.1		2	**< .001**	0.595
		A-B		**< .001**	
		A-C		**< .001**	
		B-C		NaN	

Notes: **A** - first tercile of phthalate metabolite concentration (ng/mL); **B** - second tercile of phthalate metabolite concentration (ng/mL); **C** - third tercile of phthalate metabolite concentration (ng/mL); MMP - Mono-methyl phthalate; MEP - Mono-ethyl phthalate; MiBP - Mono-isobutyl phthalate; OH-MiBP - Hydroxy-mono-isobutyl phthalate; MnBP - Mono-n-butyl phthalate; OH-MnBP - Hydroxy-mono-n-butyl phthalate; cx-MiNP - Cyclo-mono-isononyl phthalate; OH-MiNP - Hydroxy-mono-isononyl phthalate; MEHP - Mono-2-ethylhexyl phthalate; oxo-MEHP - Oxo-mono-2-ethylhexyl phthalate; OH-MEHP - Hydroxy-mono-2-ethylhexyl phthalate; cx-MEPP - Cyclo-mono-2-ethylhexyl phthalate; MBzP - Mono-benzyl phthalate; MnOP - Mono-n-octyl phthalate
