# Supplementary material for: Exploring the Association Between Sleep Patterns, Pubertal Health, and Phthalate Exposure—Preliminary Results from Slovakia
Source: Toxics. 2025 Apr 8;13(4):286. doi: 10.3390/toxics13040286 (PMC12031374; doi:10.3390/toxics13040286)
Supplement: Supplementary file 1 [file toxics-13-00286-s001.zip › Supplement 3.pdf]

Kruskal-Wallis comparison of the pubertal changes (in the stage 1-5, scaled by Tenner) to PSQI score

	χ^2		df	p	ε^2
**Thelarche**	5.58		4	0.233	0.199
		1-2		0.398	
		1-3		0.998	
		1-4		0.744	
		1-5		0.744	
		2-3		0.88	
		2-4		1	
		2-5		1	
		3-4		0.856	
		3-5		0.856	
		4-5		NaN	
**Pubarche in girls**	3.07		4	0.547	0.109
		1-2		0.983	
		1-3		0.962	
		1-4		0.996	
		1-5		0.662	
		2-3		0.979	
		2-4		0.856	
		2-5		0.619	
		3-4		0.856	
		3-5		0.856	
		4-5		0.856	
**Menarche**	3.49		4	0.48	0.129
		1-2		0.656	
		1-3		0.93	
		1-4		0.93	
		1-5		0.93	
		2-3		NaN	
		2-4		NaN	
		2-5		NaN	
		3-4		NaN	
		3-5		NaN	
		4-5		NaN	
**Genital development and pubarche in boys**	5.48		5	0.242	0.183
		1-2		0.998	
		1-3		0.884	
		1-4		0.362	
		1-5		0.41	
		2-3		0.946	
		2-4		0.522	
		2-5		0.669	
		3-4		0.843	
		3-5		0.945	
		4-5		1	
